# Graphene/MoS_2_ Nanohybrid for Biosensors

**DOI:** 10.3390/ma14030518

**Published:** 2021-01-21

**Authors:** Jinho Yoon, Joungpyo Lim, Minkyu Shin, Sang-Nam Lee, Jeong-Woo Choi

**Affiliations:** 1 Department of Chemical & Biomolecular Engineering, Sogang University, 35 Baekbeom-Ro, Mapo-Gu, Seoul 04107, Korea; moonchild@sogang.ac.kr (J.Y.); jpim92@sogang.ac.kr (J.L.); mkshin91@sogang.ac.kr (M.S.); 2 Department of Chemistry and Chemical Biology, Rutgers, The State University of New Jersey, Piscataway, NJ 08854, USA; 3 Uniance Gene Inc., 1107 Teilhard Hall, 35 Baekbeom-Ro, Mapo-Gu, Seoul 04107, Korea

**Keywords:** biosensors, graphene, transition metal dichalcogenide (TMD) nanomaterials, MoS_2_, hybrid nanomaterials

## Abstract

Graphene has been studied a lot in different scientific fields because of its unique properties, including its superior conductivity, plasmonic property, and biocompatibility. More recently, transition metal dicharcogenide (TMD) nanomaterials, beyond graphene, have been widely researched due to their exceptional properties. Among the various TMD nanomaterials, molybdenum disulfide (MoS_2_) has attracted attention in biological fields due to its excellent biocompatibility and simple steps for synthesis. Accordingly, graphene and MoS_2_ have been widely studied to be applied in the development of biosensors. Moreover, nanohybrid materials developed by hybridization of graphene and MoS_2_ have a huge potential for developing various types of outstanding biosensors, like electrochemical-, optical-, or surface-enhanced Raman spectroscopy (SERS)-based biosensors. In this review, we will focus on materials such as graphene and MoS_2_. Next, their application will be discussed with regard to the development of highly sensitive biosensors based on graphene, MoS_2_, and nanohybrid materials composed of graphene and MoS_2_. In conclusion, this review will provide interdisciplinary knowledge about graphene/MoS_2_ nanohybrids to be applied to the biomedical field, particularly biosensors.

## 1. Introduction

Ever since their unique properties were first reported, nanomaterials have been widely researched and applied in various scientific fields [1,2]. In general, as the size of the material reaches the nanometer scale, the surface area is maximized, and many beneficial properties occur that did not exist in the bulk state [3,4]. Nanomaterials are particularly promising in the field of biosensors because they can meet the criteria needed to develop highly sensitive and selective biosensors [5], an d numerous nanomaterials have been researched for this type of application [6,7]. In addition, many studies have been conducted to optimize the specific characteristics of nanomaterials, so that they can be effectively used to develop different types of biosensors, including electrochemical, fluorescent, and surface-enhanced Raman spectroscopy (SERS) biosensors [8,9,10].

Among the nanomaterials used in the development of biosensors, graphene and molybdenum disulfide (MoS_2_) are two of the most commonly studied materials being researched in recent years [11,12]. Since the discovery of fullerene, studies on carbon-based nanomaterials have been ongoing, and a myriad of novel carbon-nanomaterials have been reported, including Buckminsterfullerene (buckyball), amorphous carbon nanolayers, and carbon nanotubes (CNTs) [13,14,15]. Graphene, another type of carbon-based nanomaterial, is composed of individual carbon atomic layers arranged in a two-dimensional (2D) honeycomb lattice structure [16]. Because of its exceptional physicochemical properties, such as the large surface area, conductivity, quenching property, and easy modification, it has been frequently utilized in scientific fields ranging from biomedical to energy applications [17,18]. Various forms of graphene are already commercially available, such as graphene oxide (GO), nanographene, and reduced graphene oxide (rGO), making this nanomaterial easy to access and use. Moreover, the excellent biocompatibility of graphene makes it a promising material for biosensors and other biological applications [19,20].

In addition to graphene, transition metal dichalcogenides (TMD) are another novel type of nanomaterial that has been a popular area of research in recent years [21]. TMD nanomaterials are 2D nanomaterials composed of thin, semiconducting nanolayers of transition metal and chalcogen atoms. Because of their excellent physical, optical, and electrical properties, TMD nanomaterials are widely applied in the electronics field [22,23]. Among the different types of TMD nanomaterials, MoS_2_ has significant potential for biological applications because of its excellent biocompatibility [24]. In addition, MoS_2_ can exist in diverse structures, such as nanoparticles (NPs), nanotubes, and quantum dots (QD), and the scientific scope of their application has become broader [25,26]. Because of these properties, MoS_2_ is commonly used to develop novel types of biosensors [27,28].

**Figure 1:**
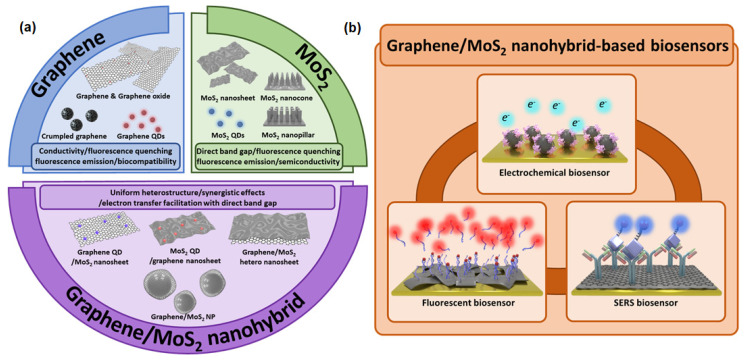
(**a**) Characteristics of graphene, molybdenum disulfide (MoS_2_), and graphene/MoS_2_ nanohybrid, and (**b**) their utilization to develop graphene/MoS_2_ nanohybrid-based biosensors.

Although both of these materials have excellent properties that are suitable for biosensors, research is now being conducted to combine these two nanomaterials to achieve a synergistic effect that exceeds the properties of each individual material [29]. Up to now, various graphene/MoS_2_ nanohybrid heterostructures have been developed for biosensor application [30,31]. Therefore, studying the characteristics of graphene, MoS_2_, and a graphene/MoS_2_ nanohybrid (Figure 1a), and discussing the usability of each material as a core component of biosensors can provide a useful foundation for developing highly sensitive and selective biosensors (Figure 1b). This review will discuss recently developed biosensors based on a graphene/MoS_2_ nanohybrid. First, the properties of graphene and MoS_2_ and their advantages in biosensors are provided. Next, their application for biosensors is discussed according to the following categories: graphene-based biosensors, MoS_2_-based biosensors, and biosensors based on a graphene/MoS_2_ nanohybrid. Overall, this review will provide interdisciplinary knowledge on the graphene/MoS_2_ nanohybrid for application in the biomedical field, particularly for the development of novel biosensors.

## 2. Graphene and MoS_2_

### 2.1. Graphene

Carbon nanomaterials are recognized as one of the most suitable candidates for diverse biological applications. Numerous studies have been conducted on stem cell therapy, differentiation, and drug delivery by utilizing the properties of carbon nanomaterials, including their outstanding mechanical, electrical, and optical properties [32,33,34,35]. In biosensor applications, CNTs, graphene, and other carbon nanomaterials have been employed to develop biosensors because of their advantages, such as excellent conductivity and easy surface modification [36]. Furthermore, depending on the structure and components of carbon nanomaterials, specific physical properties can be achieved. For example, QDs can be used for their fluorescence emission property, which broadens the potential types of carbon nanomaterial-based biosensors [37].

Since its discovery in the early 2000s, graphene has attracted huge interest for biosensing applications. Its advantages are similar to the CNT, but it has much better properties in some regards. Graphene consists of a 2D hexagonal lattice structure with individual carbon layers that have greater electrical conductivity than CNTs and copper. It also has excellent biocompatibility, flexibility, a larger surface area than other carbon nanomaterials such as CNT and graphite, and its surface can be easily modified [38,39]. The high conductivity of graphene is due to the pi (π) electrons in graphene. A carbon atom has a total of six electrons, four of which are the valence electrons in the valence shell. However, in the 2D hexagonal lattice structure of graphene, three of the four valence electrons are chemically bonded to other carbon atoms, and the remaining electron can move freely in three-dimensional (3D) space. This electron is the π electron that is located above or below the graphene sheet and has very high mobility. In certain circumstances, the π electron also behaves as if it has no mass, due to its quantum properties. As a result of these electronic properties, the conductivity of graphene is very high compared to other materials [40]. Because of these advantages, graphene is the most widely studied nanomaterial for the development of electrical/electrochemical biosensors [41,42].

**Figure 2:**
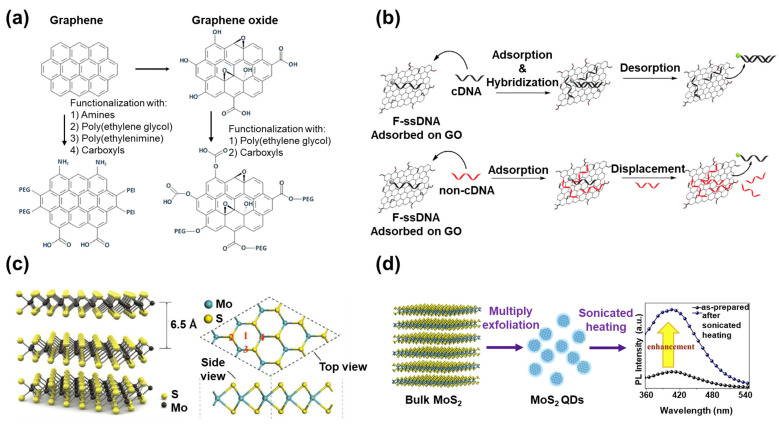
(**a**) Schematic of graphene and graphene oxide (GO) with various chemical property modification methods (reproduced with permission from [43], published by Elsevier, 2013), (**b**) the desorption of DNA from GO either via hybridization with cDNA on the surface of the GO or by nonspecific simple displacement by non-cDNA (reproduced with permission from [47], published by the American Chemical Society, 2014), (**c**) the detailed atomic structure of a MoS_2_ nanosheet (reproduced with permission from [66], published by Elsevier, 2016), and (**d**) the synthesis process of the MoS_2_ QD to improve the photoluminescence property (reproduced with permission from [69], published by Elsevier, 2015).

GO, which is the oxidized form of graphene with carboxyl and hydroxyl groups, has been frequently studied because of its easy modification using the oxidized surface chemical groups (Figure 2a). In contrast to the hydrophobicity of graphene, GO has hydrophilicity and high dispersibility in polar solvents such as water [43]. Another graphene derivative is reduced graphene oxide (rGO). Although rGO has low hydrophilicity compared to GO, it has high conductivity and a high surface area suitable for biosensing applications [44,45].

In addition to their excellent conductivity, graphene and its derivatives (GO and rGO) have much higher fluorescence quenching properties than gold, and they also have unique interactions with specific biomolecules [46]. For example, GO has a high binding affinity to single-stranded DNA (ssDNA) due to the formation of π-π interactions, so ssDNA can be easily bound to the surface of GO. However, when ssDNA forms double-stranded DNA (dsDNA) by hybridization with complementary DNA (cDNA), the binding affinity with GO decreases, and the dsDNA separates from the GO (Figure 2b) [47]. Based on these phenomena, many studies have been conducted on the development of a DNA fluorescent biosensor to diagnose genetic disorders via the conjugation of fluorescent dye-attached ssDNA with graphene and its derivatives [48]. 

Unlike the 2D structure of graphene and its derivatives, zero-dimensional (0D) graphene QDs with a similar size of less than 10 nm exhibit novel characteristics. These include extraordinary electronic properties, unique fluorescence, high solubility, and excellent photostability against photobleaching/blinking due to quantum confinement and edge effects [49,50]. In addition, other structures of graphene, such as the crumpled structure, can enhance their properties [51]. Many types of graphene have been utilized to develop outstanding biosensors based on its innate properties or conjugation with other nanomaterials.

### 2.2. MoS_2_

In addition to the studies on graphene, many other kinds of 2D nanomaterials have been studied for biological applications [52,53]. In particular, TMD nanomaterials have received notable attention as an attractive 2D nanomaterial for scientific applications including energy storage, batteries, and biological applications, because of their semimetal properties, that distinguish them from graphene [12,54,55]. For example, graphene has a band gap of 0.0 eV, similar to metal, whereas TMD nanomaterials generally have a band gap value between 0.0 eV and 3.0 eV, similar to a semiconducting material [56]. Because of these characteristics, TMD nanomaterials are especially utilized in field-effect transistor (FET)-based biosensing [57,58]. TMD nanomaterials have the structure of MX_2_, where M is a transition metal atom (Mo, W, etc.) and X is a chalcogen atom (S, Se, or Te). TMD nanomaterials have a very thin layered structure, with one layer of M atoms sandwiched between two layers composed of X atoms [59]. In addition to the layered structure, other structures of TMD nanomaterials have been studied, including the NP, nanocone, and nanopillar [60,61]. Research has also been conducted on the synthesis and application of QD-structured TMD nanomaterials with the size of several nanometers. This has been achieved through various methods such as mechanical exfoliation, intercalation-assisted exfoliation, and hydrothermal reaction [62]. In contrast to the layered structure of TMD nanomaterials, the TMD QD exhibits improved electrical and optical properties due to the quantum confinement and edge effects. TMD QDs have been widely used in the development of bioimaging platforms [63,64].

MoS_2_ nanosheet is one of the most widely studied TMD nanomaterials [65]. The thickness of a single-layered MoS_2_ is 6.5 Å, and it has a symmetric structure. It has a layer consisting of S atoms at the top and bottom and a layer consisting of Mo atoms arranged in a hexagonal shape (Figure 2c) [66]. Since there is no dangling bond on the surface, the MoS_2_ nanosheet is stable in an aqueous solution [67]. Because of its strong fluorescence quenching capability, biocompatibility, and large surface area, the MoS_2_ nanosheet is usually applied in biosensors to detect important molecules through surface functionalization with sensing probe molecules [68].

The MoS_2_ QD, with a diameter of less than 10 nm, has slightly different properties compared to the MoS_2_ nanosheet (Figure 2d) [69]. For example, as the size and thickness of the MoS_2_ nanosheet decrease, the active edge site increases. Therefore, under similar volume conditions, the MoS_2_ QD provides a larger surface area and more active sites for interaction with biomolecules than the layered structure of MoS_2_. In addition, since the increased active edge site improves the current flow density, the MoS_2_ QD has huge potential as a high-functionality electrocatalyst [70]. In the field of biosensing, the MoS_2_ QD has been frequently used for bioimaging and labeling as a fluorescence probe due to its easy synthesis, high photostability, and controllable fluorescence emission properties [71,72]. As demonstrated by these examples, MoS_2_ has been synthesized in multiple structures with unique properties to develop various types of biosensors.

Recently, research has been conducted on combining graphene and MoS_2_ to develop biosensors with a synergistic effect that exceeds the properties of each nanomaterial individually. The developed structure of a nanohybrid composed of graphene and MoS_2_ is decided depending on its applied purpose. If the purpose of application is the development of wearable or flexible biosensors, thin nanolayered structures are required to retain its structure after numerous bending processes. Like this example, although there are studies that claim that MoS_2_ possesses biocompatibility, other reports insist that MoS_2_ is somewhat toxic to living things [73,74,75]. However, by encapsulating the MoS_2_ in graphene, MoS_2_ can achieve a more certain biocompatibility, which can be applied to in vivo research. Moreover, although graphene is also an excellent conductive material by itself, when combined with MoS_2_, even if the basic properties are similar, and the electron transport improvement and electrochemical signal enhancement become greater than those of graphene alone. Besides, the graphene/MoS_2_ nanohybrid can preserve the effective carrier mobility of graphene while facilitating the electron transfer. This property is suitable for enhancing the redox signals in the biosensing process to achieve high sensitivity [30]. In addition, by combining the specific structures of graphene and MoS_2_, a novel biosensing platform can be developed. For example, by combining MoS_2_ QDs and graphene, or graphene QDs and MoS_2_, different types of fluorescent biosensing systems can be developed that are appropriate for target molecules and environmental conditions [76,77]. Although covalent bonds are not yet used for the bonding of graphene and MoS_2_ in most studies, in order to form more efficient and stronger bonds in nanohybrid and stabilize the structures, there were studies that used covalent bonds to develop the nanohybrid or modified the surface of each nanomaterial covalently [78,79,80,81].

## 3. Graphene in Biosensors

As described in Section 2.1, graphene and its derivatives (GO, rGO, and graphene QD) have properties such as electrochemical signal amplification, fluorescence emission, and surface-enhanced Raman scattering activity, which have been widely applied in biosensing [82,83,84]. In addition, since the surface chemical property can be easily modified, it is easy to immobilize various biomolecules, such as nucleic acids, enzymes, and antibodies, on graphene. These biomolecules can be used as the sensing probes to detect target biomolecules [85,86,87]. Because of these advantageous properties, many studies are underway on the fabrication of biosensors using graphene and its derivatives. 

**Figure 3:**
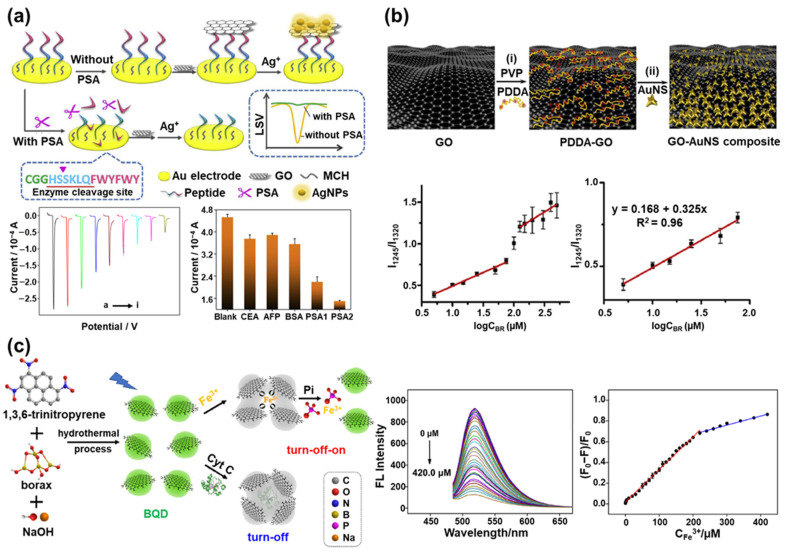
Graphene in biosensors: (**a**) Schematic of the electrochemical prostate-specific antigen (PSA) biosensor based on the GO/AgNP hybrid through the peptide cleavage strategy and a linear sweep voltammogram for PSA detection (Reprinted with permission from [88], published by Elsevier, 2019), (**b**) Schematic illustration of the synthesis process of AuNS on the surface of the GO and label-free detection of the bilirubin (Reprinted with permission from [96], published by Elsevier, 2019), (**c**) Schematic illustration of the boron-doped graphene QD (BQD) synthesis process for detecting Fe^3+^, Cyt C, and Pi and the fluorescence signal of BQD with the addition of a different concentration of Fe^3+^ and its linear response curve (Reprinted with permission from [104], published by Elsevier, 2019).

An electrochemical prostate-specific antigen (PSA) biosensor based on the hybridization of GO and silver nanoparticles (AgNPs) was developed by introducing the peptide cleavage strategy (Figure 3a) [88]. The PSA, a 33 kDa, single-chain glycoprotein, which is produced by cells in the prostate gland, exhibits a protease activity similar to chymotrypsin. PSA generally exists in human serum at a concentration of 4 ng/mL or less [89,90]. However, if PSA exceeds this concentration, it is an indicator for prostatic diseases such as prostate cancer, prostatitis, and prostatic hyperplasia [91]. For this reason, PSA is an important biomarker for the diagnosis of prostatic diseases, and the accurate measurement of PSA concentration in the blood is very important for the effective treatment of prostatic diseases. To develop this PSA biosensor, peptides with a specific sequence (CGGHSSKLQFWYFWY) were first immobilized on the Au substrate, which was used as the sensing probe to be degraded in the presence of PSA. Then, GO and the AgNPs derived from silver nitrate (AgNO_3_) were bound to the immobilized peptides on the Au substrate sequentially. The aromatic amino acids of peptides act as a chemical link, so the GO was bound to peptides through π-π interaction. Moreover, due to the reduction reaction between the GO and Ag^+^, the AgNP was stably immobilized on the GO. Then, PSA was introduced as the protease by utilizing the peptide cleavage property of PSA. In the presence of the PSA, the GO and AgNPs bound to the peptides were removed from the Au substrate because of the degradation of the peptides by the PSA. Therefore, electrochemical signals derived from the AgNPs decreased proportionally to the concentration of PSA. In the absence of PSA, the GO and AgNPs still exist on the Au substrate, and the electrochemical signals derived from AgNP can be measured with high sensitivity through the electrochemical signal-enhancing effect of GO. Using this sensing mechanism, the developed PSA biosensor showed high sensitivity (detection limit of 0.33 pg/mL) for detecting PSA prepared in clinical serum samples. In a similar study, DNA and electroactive Prussian blue was utilized instead of the peptide sequences and the AgNPs. This electrochemical microRNA-122 biosensor was developed using the excellent conductivity of GO [92]. In addition, an enzymatic electrochemical biosensor was developed based on a hybrid layer composed of CNTs and GO to detect lactate [93].

In addition to electrochemical biosensors, graphene and GO have been widely employed to develop SERS-based biosensors due to the graphene-enhanced Raman scattering [94]. For example, an anisotropic gold-copper alloy covered with GO was proposed to develop a SERS-based biosensor to detect apurinic/apyrimidinic endonuclease 1 [95]. In another example, a paper-based SERS biosensor using GO and gold nanostars (AuNSs) was developed for the label-free detection of bilirubin (Figure 3b) [96]. Since the abnormal concentration of bilirubin in the serum causes hyperbilirubinemia-related diseases such as jaundice, cholelithiasis, and cholangitis, it is important to accurately measure the concentration of bilirubin, especially in young babies [97,98]. This biosensor was fabricated by placing a hybrid composite of GO and AuNSs on the filter paper. The AuNSs, which exhibit a remarkably high SERS-enhancing effect, were introduced because they provide inherent SERS hot-spots without a complicated manipulation process. GO was introduced because of its SERS signal-enhancing property, large surface area, and efficient binding with bilirubin. The bilirubin could be captured on the GO through the electrostatic and π-π interaction. There was no need to introduce Raman dyes because the Raman signal of bilirubin itself was used for SERS biosensing. Thus, the rapid and sensitive detection of bilirubin could be achieved by SERS without the Raman dye tagging process. This SERS biosensor exhibited excellent bilirubin sensing performance with a linear response in two ranges of 5.0–150 μM and 150–500 μM, with a limit of detection (LOD) of 0.436 μM.

Fluorescent biosensors have been studied extensively because of their easy operation and rapid detection characteristics, which are suitable for point-of-care-testing (POCT) applications [99,100,101]. To develop graphene-based fluorescent biosensors, the graphene QD’s own fluorescence emission property and the quenching property of the graphene are often utilized [102,103]. For example, boron-doped graphene QD (BQD) was synthesized to develop a fluorescent biosensor to detect Fe^3+^, cytochrome C (Cyt C), and phosphate (Pi), as shown in Figure 3c [104]. Fe^3+^ is an important ion that acts as a co-cofactor in metalloproteins, and it is also known to cause neurodegenerative diseases and organ dysfunction when it is present in excess in humans [105]. Pi can also cause serious problems with physical activity when present in excessive amounts [106]. 

In this study, the quantum yield of synthesized BQD was improved up to 16.8% by doping boron, and it enabled the selective detection of Fe^3+^. The BQD had a maximum excitation wavelength of 480 nm and a maximum emission wavelength of 520 nm. The fluorescence quenching occurred when Fe^3+^ was added because Fe^3+^ can induce the aggregation of BQD through the coordination of abundant oxygen groups of the BQD, resulting in fluorescence quenching. Therefore, the intensity of fluorescence emitted from BQD decreased in proportion to the concentration of added Fe^3+^. The fabricated BQD-based biosensor measured the Fe^3+^ concentration linearly in two ranges, from 50 nM to 220 μM and from 220 μM to 420 μM, with a detection limit of 31.2 nM. This biosensor also succeeded in the selective detection of Cyt C, since Cyt C possesses Fe^3+^. Cyt C was measured linearly in two ranges, from 10 μg/mL to 300 μg/mL and 300 μg/mL to 2000 μg/mL, with a detection limit of 5.9 μg/mL. After inducing the fluorescence quenching by detecting Fe^3+^, this fluorescence-quenched biosensor was used to detect Pi. Since Fe^3+^ has a greater affinity to the oxygen donor atom of Pi than the oxygen group of BQD, the aggregated BQD dissociates and recovers the fluorescence emission when Pi is added. Using this two-step sensing mechanism, the fluorescence of BQD is first quenched by the detection of Fe^3+^ and then restored in proportion to the concentration of added Pi. By measuring the restored fluorescence intensity of the BQD, Pi was linearly measured in the range of 3 μM to 40 μM, and the detection limit was 340 nM. This biosensor suggested the novel method of detecting multiplexed target molecules sequentially using the fluorescent emission property of graphene QDs. 

Another study reported a fluorescence resonance energy transfer (FRET) biosensor to detect *Staphylococcus aureus*-specific DNA [107]. This biosensor detected target DNA based on fluorescence quenching via the FRET effect caused by the proximity of AuNPs and graphene QDs. This was achieved by co-hybridizing the target DNA with the reporter gene immobilized on the AuNP and the capturing gene immobilized on the graphene QDs. In the presence of target DNA, the reporter gene on the AuNP and the capturing gene on the graphene QDs can hybridize with each different half of the whole target DNA sequence, and then the FRET effect occurred due to the connection between the AuNPs and the graphene QDs via target DNA detection. 

The development of a label-free bleomycin biosensor has also been reported using a mechanism of recovering the fluorescence signal of nitrogen-doped graphene QDs, which are quenched by ssDNA immobilized on their surface through the decomposition of ssDNA in the presence of bleomycin [108]. All of the described graphene QD-based biosensors were capable of detecting target molecules sensitively due to the outstanding fluorescence property of graphene QDs. 

In addition to graphene QDs, the quenching property of the graphene itself has been used for sensitive fluorescent biosensing. For example, a bisphenol A (BPA) biosensor based on magnetic oxidation graphene (MGO) and fluorescein-labeled anti-BPA aptamer (BSA aptamer) was developed [109]. The MGO was conjugated with fluorescein-labeled anti-BPA aptamer to form a MGO/BSA aptamer complex. Due to the fluorescence quenching property of the MGO, the MGO/BSA aptamer complex cannot emit a fluorescence signal. However, in the presence of BPA, since the BSA aptamer prefers to combine with BPA rather than MGO, the BSA aptamer detaches from the MGO and forms the BSA/BSA aptamer complex. This leads to a recovered fluorescence signal proportional to the added BSA concentration. To improve the sensing sensitivity, the MGO that detached from the BSA aptamer was completely removed by magnetic separation. In another study, a universal fluorescence sensing platform for the detection of target DNA was developed using GO as a fluorescence quencher [110]. The base sequence of the target DNA detected on this platform can be designed as a desired sequence of a specific disease, bacteria, or virus. Similar to the sensing mechanism of the previously mentioned biosensor, the fluorescent dye-modified ssDNA, which was capable of hybridizing with the target DNA, was bound to the GO. When the target DNA was added, the ssDNA separated from the GO by forming dsDNA restored the fluorescence signal proportional to the concentration of the target DNA. It is notable that the sensitivity of the biosensor was greatly increased by using the reduced graphene QD as the fluorescent molecule conjugated to ssDNA. This study suggested a new method to develop an excellent fluorescent platform by utilizing the two different types of graphene simultaneously. 

In addition to the research discussed here, various types of graphene and graphene derivative-based biosensors have been reported for a broad range of applications in the medical, pharmaceutical, environmental, and food industries [111,112,113]. As seen in this section, excellent biosensors are being developed utilizing the unique properties of graphene and its derivatives. Although graphene has a huge potential for developing biosensors, it also has some limitations, such as the lack of band gap. To overcome the limitations of graphene, research is underway to develop a heterostructure composed of graphene and other nanomaterials. These studies will contribute to the development of exceptional graphene-based biosensors with ultra-sensitivity, beyond the studies discussed here. 

## 4. MoS_2_ in Biosensors

MoS_2_ often forms a heterostructure with other nanomaterials, and it is widely applied in the development of biosensors using a variety of detection techniques, such as electrochemical, electrochemiluminescence, fluorescence, colorimetric, and SERS [114,115,116,117,118]. MoS_2_ is also being synthesized in structures including nanosheets, NPs, QDs, and nanorods to maximize the sensing efficiency of target molecules [25,26,119,120]. Since it is simple to modify the surface of MoS_2_, the biomolecules capable of binding with target molecules can be easily immobilized on the surface of MoS_2_. Because of these properties, many MoS_2_-based biosensors have been studied and reported recently [27,121]. 

**Figure 4:**
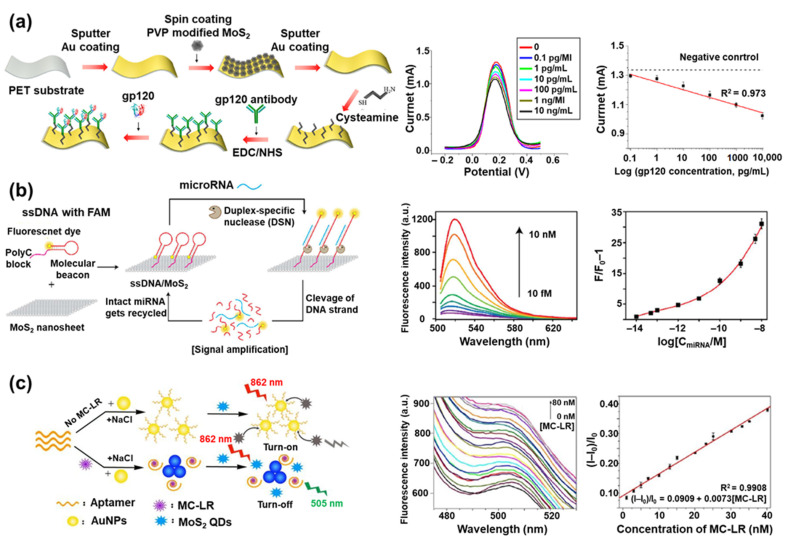
MoS_2_ in biosensors: (**a**) Schematic illustration of the fabrication process of the HIV biosensor based on an Au/MoS_2_/Au multilayer on a polyethylene terephthalate (PET) substrate; the square wave voltammogram for detecting gp120 with its linear response curve (Reprinted with permission from [122], published by MDPI, 2019), (**b**) Schematic illustration of the let-7b miRNA detection mechanism using a fluorescent biosensor composed of the MoS_2_ nanosheet, ssDNA with fluorescein amidite (FAM), and duplex-specific nuclease (DSN); the measured fluorescence signal with different concentrations of the let-7b miRNA and its linear response curve (Reprinted with permission from [125], published by American Chemical Society, 2018), (**c**) Schematic illustration of the MC–LR detection process using MoS_2_ quantum dots (QDs) and aptamer-modified AuNPs; the fluorescence signals of MoS_2_ QDs by with different added concentrations of microcystin–LR (MC–LR) with its linear response curve (Reprinted with permission from [130], published by American Chemical Society, 2020).

One example of a MoS_2_-based electrochemical biosensor is a human immunodeficiency virus (HIV) biosensor with a multi-layered structure composed of MoS_2_ NPs between two Au nanolayers (Au/MoS_2_/Au multilayer) on a polyethylene terephthalate (PET) substrate [122]. To develop this biosensor, the authors targeted the envelope glycoprotein GP120 (gp120), which is a glycoprotein on the surface of HIV, and the gp120 antibodies were immobilized on the Au/MoS_2_/Au multilayer to detect gp120 (Figure 4a). The detection of gp120 was carried out in a [Fe(CN)_6_]^3−/4−^-containing solution with square wave voltammetry. The [Fe(CN)_6_]^3−/4−^ was used as a redox probe and exhibited a current value through the electron transfer process with the surface of the HIV biosensor. When the gp120 was added and combined with the gp120 antibody on the Au/MoS_2_/Au multilayer, the redox signal from [Fe(CN)_6_]^3−/4−^ was blocked, decreasing the measured current due to the nonconductivity of the captured gp120. The decreasing current value was linearly proportional to the added gp120 concentration, enabling the sensitive detection of gp120 concentration. Using this HIV biosensor, the gp120 was measured with a linear response in a broad concentration range of 0.1 pg/mL to 10 ng/mL. Because of the effective electron transfer and large surface area of the MoS_2_ NPs, it showed higher current values compared to the normal Au substrate prepared without MoS_2_ NPs. Based on the detection limit of 0.066 pg/mL, it was proved that gp120 was measured more sensitively than in other electrochemical HIV biosensors previously reported. 

In addition to this study, an electrochemical lactate biosensor was developed using a MoS_2_ nanosheet and lactate oxidase (LOx) [123]. To investigate the size effect of the MoS_2_ nanosheet for enhancing the electrochemical properties, various sizes of the MoS_2_ nanosheet were prepared through exfoliation, and a 90-nm MoS_2_ nanosheet was chosen as the optimal size for fabricating the lactate biosensor. The lactate was measured in a linear concentration range of 0.056 mM to 0.77 mM with high sensitivity (6.2 μA·mM^−1^), an excellent detection limit (17 μM), and good reproducibility (relative standard deviation (RSD) value of 4.7%). 

In another example, an electrochemical biosensor based on a poly-xanthurenic acid (PXA)-functionalized MoS_2_ nanosheet was reported for detecting the PIK3CA gene, which is related to gastric carcinoma [124]. The xanthurenic acid was electropolymerized on the surface of the MoS_2_ nanosheet, forming PXA/MoS_2_. On the surface of the PXA/MoS_2_, ssDNA was immobilized through π-π interaction between the nucleobases of ssDNA and PXA. While measuring the interfacial impedance values by electrochemical impedance spectroscopy, the interfacial electron transfer resistance (R_ET_) value increased when the ssDNA was immobilized on the PXA/MoS_2_ due to the nonconductivity of the ssDNA. However, when the PIK3CA gene was added, it hybridized with the ssDNA and formed dsDNA. The dsDNA was released from the PXA/MoS_2_ because the binding affinity between ssDNA and the PIK3CA gene was higher than the π-π interaction between the ssDNA and PXA. Because of the release of ssDNA from PXA/MoS_2_, the R_ET_ value increased in the presence of the PIK3CA gene. Using this biosensor, the PIK3CA gene could be measured linearly in the concentration range of 1.0 × 10^−16^ mol/L to 1.0 × 10^−10^ mol/L, with a detection limit of 1.8 × 10^−17^ mol/L. 

MoS_2_ nanosheets are often used to develop fluorescent biosensors by taking advantage of their distinct optical property. For instance, a fluorescent let-7b microRNA (miRNA) biosensor was developed by combining MoS_2_ nanosheets, ssDNA with a fluorescent dye (fluorescein amidite, FAM), and duplex-specific nuclease (DSN) (Figure 4b) [125]. The family of let-7 miRNAs is important because it is known to be deeply involved in the differentiation process of cells in the human body, and when mis-regulation occurs, these miRNAs can cause diseases such as cancer [126]. In particular, let-7b miRNA is used as a biomarker for diagnosing breast cancer [127]. In the ssDNA introduced as a sensing probe, one end of the ssDNA was modified with a FAM and the other end was designed to form a poly-cytosine sequence (poly C). Because of poly C, the ssDNA could be immobilized on the MoS_2_ nanosheet by a van der Waals force without any chemical linker. After immobilization on the MoS_2_ nanosheet, the fluorescence signal emitted from the FAM of the ssDNA was quenched due to the fluorescence quenching property of the MoS_2_ nanosheet. However, in the presence of let-7b miRNA, the ssDNA could hybridize with the let-7b miRNA to form a DNA/RNA heteroduplex structure. This structure would then be decomposed by DSN, which can selectively cleave the DNA in a DNA/RNA heteroduplex structure. Lastly, the fluorescence signal was restored after let-7b miRNA detection. During the decomposition of the DNA/RNA heteroduplex structure by DSN, the let-7b miRNA maintained its original structure, unlike the ssDNA which was degraded in small base pairs. Because of this property, the let-7b miRNA that separated from the DNA/RNA heteroduplex structure could be reacted again with the unreacted ssDNA immobilized on the MoS_2_ nanosheet. This induced the amplification of the fluorescence signal similar to the polymerase chain reaction (PCR). This biosensor measured let-7b miRNA in a linear response range of 10 fM to 10 pM with a LOD of 3.4 fM. 

Another study developed a silver ion (Ag^+^) biosensor composed of a MoS_2_ nanosheet and FITC-attached ssDNA (FITC–ssDNA) [128]. The Ag^+^ had the property of forming a stable C–Ag^+^–C mismatched base pair by selectively binding to the C of DNA. Therefore, when Ag^+^ was added to fluorescence-quenched FITC–ssDNA on the MoS_2_ nanosheet, the FITC–ssDNA in the conformation of a random coil structure changed its shape into a double helix structure due to the formation of the C–Ag^+^–C mismatched base pair. As FITC–ssDNA formed a double helix structure, the binding force between the FITC–ssDNA and MoS_2_ nanosheet decreased, causing the release of the FITC–ssDNA from the MoS_2_ nanosheet and restoring fluorescence. The Ag^+^ concentrations were measured in a linear range of 1 nM to 100 nM with a LOD of 1nM using this biosensor. In another study, a biosensor to detect ochratoxin A was developed based on MoS_2_ nanosheets as a fluorescence quencher and a semiconductor QD-conjugated ochratoxin A aptamer as the fluorescence probe [129]. 

In contrast to the fluorescence quenching property of the MoS_2_ nanosheet, the fluorescence emission property of the MoS_2_ QD has been utilized to develop fluorescent biosensors. For example, a microcystin–LR (MC–LR) biosensor was developed using the inner filter effect (IFE) between MoS_2_ QDs and aptamer-modified AuNPs (Figure 4c) [130]. MC–LR strongly inhibits the activity of protein phosphatases, causing liver damage, and the risk of tumors increases significantly, even when exposed to minimal amounts of MC–LR [131]. Therefore, it is very important to sensitively detect MC–LR. To achieve this, MoS_2_ QDs with upconversion fluorescence were synthesized by introducing *N*-acetyl-l-cysteine (NAC), which acted as a capping agent during the synthesis process. The upconversion fluorescence occurred through two successive energy transfer processes between the NAC and MoS_2_ QD, with effective elimination of background interference. When the aptamer-modified AuNP presented with the MoS_2_ QD, IFE was induced due to the plasmon absorption characteristics of the aptamer-modified AuNP, and the fluorescence signal of the MoS_2_ QD was quenched. However, in the presence of MC–LR, the MC–LR combined with the aptamer on the AuNP and significantly aggregated the aptamer-modified AuNPs. Through the aggregation of AuNPs, IFE was hindered and the fluorescence signal of the MoS_2_ QD was recovered. Using this sensing mechanism, MC–LR was measured in a broad linear concentration range of 0.05 nM to 40.19 nM with a detection limit of 0.01 nM. 

In addition, an ascorbic acid biosensor was developed using the MoS_2_ QD and MnO_2_ nanosheet [132]. In this study, the MoS_2_ QD and MnO_2_ nanosheet were utilized as the fluorescence molecule and quenching material, respectively. In the presence of ascorbic acid, the quenched fluorescence signal of the MoS_2_ QD recovered through the oxidization of the MnO_2_ nanosheet by the ascorbic acid. The concentration of the ascorbic acid was measured in a range of 0.33 μmol/L to 5.00 μmol/L with a LOD of 39 nmol/L, based on the degree of fluorescence signal recovery, that was proportional to the concentration of ascorbic acid.

The pH insensitive fluorescence emission property of the MoS_2_ QD has been applied to develop a urea biosensor [133]. To detect urea, 2,3-diaminophenazine (DAP), which is a pH-sensitive fluorescent molecule, and urease for enzymatic reaction with urea were utilized in this biosensor. The urease catalyzed the hydrolysis of urea to produce ammonia, making the solution basic. Since the fluorescence signal of DAP becomes stronger as pH increases, the concentration of urea could be measured in the range of 5 μM to 700 μM by analyzing the fluorescence intensity ratio of the DAP and MoS_2_ QDs. This ratiometric fluorescence analysis using the comparison between DAP and MoS_2_ QDs was used because the fluorescence instability of DAP can be significantly affected by environmental conditions, which hinders accurate measurement. Therefore, through ratiometric fluorescence measurement using MoS_2_ QDs as the reference standard for fluorescence intensity, urea could be measured based on the pH change of the solution in real-time.

As demonstrated by these studies, MoS_2_ has been widely applied to develop different types of biosensors using its unique electrochemical and optical properties [134,135]. MoS_2_ has also been recently researched for use in highly sensitive SERS biosensors [136,137,138]. Although MoS_2_ has many benefits for biosensors, its limitations include having a relatively low conductivity and Young’s modulus compared to graphene [139]. For these reasons, there are efforts to create heterostructures with MoS_2_ and graphene to achieve improved sensing performance through the synergistic effect of improving electron transfer and forming a wide band gap [140].

## 5. Graphene/MoS_2_ Nanohybrid in Biosensors

As seen in the previous sections, graphene and MoS_2_ have been frequently utilized to develop advanced biosensors. When these two novel nanomaterials are combined, the properties derived from each material are enhanced, and synergistic effects can be achieved to produce outstanding biosensors with ultra-sensitivity and selectivity compared to biosensors that use each nanomaterial separately. This section discusses recently reported biosensors based on a graphene/MoS_2_ nanohybrid. 

**Figure 5:**
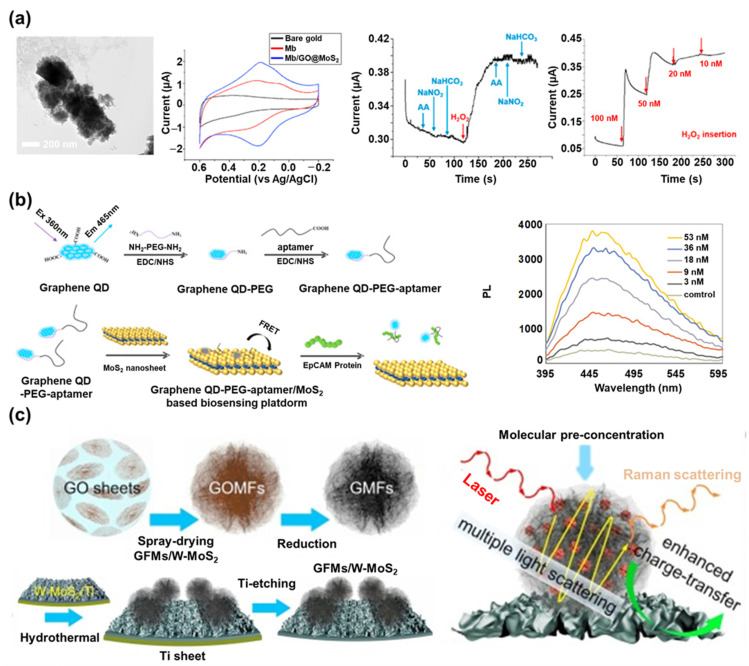
Graphene/MoS_2_ nanohybrid in biosensors: (**a**) TEM image of the MoS_2_ nanoparticles (NPs) encapsulated by GO, cyclic voltammogram of Mb modified GO@MoS_2_, and its selective and sensitive H_2_O_2_ sensing performance by amperometric I-T measurement (reproduced with permission from [30], published by Elsevier, 2017), (**b**) schematic diagram of the fluorescent epithelial cell adhesion molecule (EpCAM) biosensor based on the graphene QD and MoS_2_ nanosheet, and its fluorescent sensing of EpCAM depending on the concentration of EpCAM (reproduced with permission from [76], published by Elsevier, 2017), (**c**) schematic images of W-MoS_2_ nanosheet decorated with graphene micro-flowers (GMFs) fabrication and its surface-enhanced Raman spectroscopy (SERS)-enhancing effects for target detection (reproduced with permission from [77], published by Elsevier, 2020).

Recently, Choi’s group reported a graphene/MoS_2_ nanohybrid-based electrochemical biosensor in which MoS_2_ NPs were encapsulated by GO (Figure 5a) [30]. The MoS_2_ NPs were used to extend the activated surface area of the MoS_2,_ and GO was used to develop the spherical structure. Since GO has functional groups on its surface and there is a minimal surface modification process, GO is easier to combine with MoS_2_ than graphene. To create this nanohybrid, the surface of the MoS_2_ NPs was modified with the amine group via chemical linkers and encapsulated by GO through the electrostatic bond (GO@MoS_2_). Next, myoglobin (Mb) was employed on the GO@MoS_2_ nanohybrid as the sensing probe to detect H_2_O_2_ through the electrochemical reaction using the iron ions located in its structure. The proposed GO@MoS_2_ nanohybrid provided the biocompatible, extended surface area for the Mb immobilization and facilitated the electron transfer reaction that occurred at the electrode surface. This biosensor exhibited enhanced electrochemical signals from the Mb based on cyclic voltammetry (CV) analysis, and it showed excellent H_2_O_2_ sensing sensitivity (20 nM concentration level) and selectivity based on an amperometric I-T investigation when combined with ascorbic acid, NaNO_2_, and NaHCO_3_. In addition, it could retain its sensing property for 9 days because of the biocompatible property of the GO@MoS_2_ nanohybrid. This nanohybrid-based biosensor exhibited an enhanced redox signal as −1.86 μA at an oxidation potential and 1.95 μA at a reduction potential compared to the biosensors prepared without nanohybrid (−1.04 μA at an oxidation potential and 1.12 μA at a reduction potential), and only MoS_2_ or only GO. In addition, its detection limit value (20 nM) was much more sensitive than the results of the other studies, like AuNP- (500 nM), carbon/AgNP- (50 nM), or graphene-based (340 nM) electrochemical H_2_O_2_ biosensors [141,142,143]. This result verified the effect of the hybridization of graphene and MoS_2_. Beyond this study, this group also reported a more efficient method to develop the graphene/MoS_2_ nanohybrid for biosensor application [31]. During the synthesis process, a direct surface modification of the MoS_2_ NPs was conducted to remove the surface modification of the MoS_2_ NPs using complex chemical linkers that could perform the capacitance role between the MoS_2_ and GO. Synthesized amine-modified MoS_2_ NPs were efficiently encapsulated by GO, and the newly developed graphene/MoS_2_ nanohybrid exhibited more enhanced redox signals derived from the Mb than the previous study. 

In addition to these studies, many electrochemical biosensors have been developed using the graphene/MoS_2_ nanohybrid, such as the 3D graphene/MoS_2_ aerogel and rGO decorated with MoS_2_ QDs [144,145]. In one research, the graphene/MoS_2_ nanohybrid composed of nanoflower-structured MoS_2_ and graphene was combined with AuNPs and modified on a glassy carbon electrode (AuNPs/MoS_2_/GN). This proposed nanohybrid showed excellent electro-oxidative activity with the nitrite ions to develop an electrochemical nitrite ion biosensor with wide a linear response from 5 μM to 5 mM and detection limit of 1 μM [146]. In addition to this example, a MoS_2_ nanosheet decorated with graphene QDs was reported for developing an electrochemical mycotoxin biosensor [147].

In addition to electrochemical biosensors, the graphene/MoS_2_ nanohybrid is also being used for fluorescent biosensors. In one study, a fluorescent biosensor based on the graphene QD and MoS_2_ nanosheet was developed to detect the protein EpCAM, an important epithelial cell adhesion molecule [76]. The fluorescent signal of the graphene QD was used as the sensing signal, and the MoS_2_ nanosheet was used as the quenching material (Figure 5b). To fabricate the sensing probe for EpCAM, the carboxyl-modified EpCAM aptamer was modified on the amine-modified graphene QD via EDC/NHS bonding, and the prepared sensing probe was immobilized on the MoS_2_ nanosheet. In the absence of EpCAM, the fluorescent signal of the graphene QD was quenched by the MoS_2_ nanosheet. However, in the presence of EpCAM, the sensing probe composed of graphene QDs and EpCAM aptamers detached from the MoS_2_ nanosheet due to the strong binding affinity between the aptamer and EpCAM. The fluorescent signal of the graphene QDs was restored after the detection of EpCAM. This graphene/MoS_2_ nanohybrid biosensor exhibited a highly sensitive detection of EpCAM with a LOD of 450 pM, and a linear detection range of 3 nM to 54 nM, whose sensitivity was comparable to the other techniques-based EpCAM biosensors, including immunosensors (100 pg amount and 200 pg/mL), and much more sensitive than the other fluorescent EpCAM biosensor (1 ng/mL) [148,149,150].

A different study created a highly sensitive chemiluminescent biosensor using a graphene/MoS_2_ nanohybrid for cholesterol detection [151]. In this study, the graphene QD and MoS_2_ QD were employed simultaneously to catalyze the maximum chemiluminescence emission of rhodamine B, which was used as the chemiluminescence sensing signal molecule. Cholesterol oxidase was used as the sensing probe to detect cholesterol. After the enzymatic reaction between cholesterol oxidase and cholesterol, the generated H_2_O_2_ reacted with the rhodamine B to emit the strong chemiluminescence signal. At this point, the graphene QD and MoS_2_ QD accelerated the enzymatic reaction and induced an enhanced chemiluminescence emission to achieve high sensitivity (Detection limit: 35 nM) compared to the other techniques- or nanomaterials-based cholesterol biosensors, including the electrochemical titanium/AuNP-based biosensor (13 µM), chemiluminescence CuO NP-based biosensor (170 nM), and colorimetric graphene QD-based biosensor (6 µM) [152,153,154].

Beyond the application of simple target molecule detection, there was a study that used a graphene/MoS_2_ nanohybrid to monitor circulating tumor cells [155]. It is likely that a graphene/MoS_2_ nanohybrid could be used for sensing more complex substances or monitoring cellular states such as cell differentiation or cancer cell monitoring.

The graphene/MoS_2_ nanohybrid has also been used to develop surface plasmon resonance (SPR) biosensors and SERS biosensors [156,157,158]. For example, an optical fiber-based SPR biosensor composed of Ag, MoS_2_, and a graphene nanohybrid was developed to sense DNA hybridization through computational modeling [159]. By introducing a single MoS_2_ nanolayer between the Ag and graphene layer, the sensitivity of the SPR biosensor increased because of the absorption and optical ability of graphene and the quenching effect of MoS_2_. Based on the mathematical modeling results, this proposed biosensor had a high sensitivity of 105.71 deg/RIU, excellent refractive index sensitivity, and detection accuracy for target DNA sequences. The sensitivity of this biosensor was much higher than the sensitivity of graphene-based SPR biosensors (Sensitivity range: 86.43~94.29 deg/RIU) and MoS2-based SPR biosensor (53.49 deg/RIU).

In another example, an SPR biosensor was proposed using a hybrid layer composed of aluminum and a graphene/MoS_2_ nanohybrid to achieve high sensitivity [160]. The hetero-structured layers composed of MoS_2_/aluminum/MoS_2_/graphene were developed as the highly sensitive SPR substrate. The numbers of MoS_2_ layers were optimized to maximize the sensitivity (190.83 deg/RIU). 

Similar to SPR biosensors, the graphene/MoS_2_ nanohybrid has been studied for use as a SERS-sensitive substrate for biosensing applications. For instance, a nano-mesh structure composed of GO, MoS_2_, and AuNPs was fabricated on a 3D nickel substrate to form highly dense SERS hot spots to develop a SERS biosensor [161]. In this study, the graphene/MoS_2_ nanohybrid induced the chemical enhancement effect of the SERS signal, and it could provide the nanogap spots in which AuNPs were selectively inserted without an aggregation problem. Moreover, a 3D nickel substrate was used as the template where the graphene/MoS_2_ nanohybrid with AuNPs was spin-coated to make the SERS-sensitive substrate, and the 3D nickel had the Raman signal enhancement effect. To verify the SERS sensitivity of this substrate, Raman-active molecules such as crystal violet (CV) and rhodamine 6 G (R6G) were introduced onto the prepared substrate. Based on the results, the biosensor could detect CV and R6G with remarkably high sensitivity (LOD of 10^−14^ M). In addition, because of the flexibility of the 3D nickel substrate, the SERS-sensitive substrate retained its sensing property after bending, making it a potentially versatile SERS platform applicable for in-situ detection on any rough surface. In another study, a wrinkled MoS_2_ (W-MoS_2_) nanosheet decorated with graphene micro-flowers (GMFs) was developed for SERS biosensing application (Figure 5c) [77]. The flower structures of the GMFs provided abundant SERS hot spots and an effective pre-concentration process of target molecules, and the W-MoS_2_ provided a large activated surface area. By combining these nanomaterials with unique structures, a synergistic effect was generated through the enhanced layer-by-layer interactions. To achieve this, the spray-drying method was used to prepare the GO micro-flowers, and a reduction process was conducted to prepare the GMFs. Next, W-MoS_2_ was synthesized on the Ti sheet, and the GMFs were modified on the synthesized W-MoS_2_ layer. In the final step, the Ti sheet, which was used as the template, was removed by an acidic solution. The prepared graphene/MoS_2_ nanohybrid induced a highly enhanced SERS sensitivity because of the effective pre-concentration process and the enhanced charge transfer and multiple light scattering effects derived from the W-MoS_2_ and GMFs hybrid. To evaluate the SERS signal enhancing effect of this substrate, rhodamine B, methylene blue, and adenosine were employed as the Raman probe molecules on the substrate. The proposed SERS substrate successfully detected the Raman probe molecules with high sensitivity (LOD for rhodamine B was 5 × 10^−11^ M). The proposed graphene/MoS_2_ nanohybrid showed a much better sensitivity than the other recently reported nanomaterial-based SERS substrates, such as graphene QD (1 × 10^−9^ M) and MoS_2_ (1 × 10^−7^ M) [162,163].

As discussed in this section, the graphene/MoS_2_ nanohybrid is being utilized to develop outstanding biosensors with high sensitivity and selectivity. In addition, there are some researchers trying to utilize the graphene/MoS_2_ nanohybrid to develop wearable or flexible biosensors and electronic devices [164,165,166]. Through this research, the graphene/MoS_2_ nanohybrid is expected to play a role as a key component in the development of next-generation biosensors. All of the studies discussed in detail in this section are summarized in Table 1.

## 6. Conclusions and the Future Perspective

Since nanomaterials were first discovered, numerous nanomaterials have been synthesized, researched, and applied in many scientific fields because of their unique properties. Among the many nanomaterials, graphene and MoS_2_ are particularly promising nanomaterials for highly sensitive and selective biosensors. Graphene, with 2D single carbon atomic layers arranged in a honeycomb lattice structure, possesses the most exceptional properties among numerous carbon-based nanomaterials, such as high conductivity, biocompatibility, and large surface area. TMD nanomaterials have also been studied recently because of their direct bandgap, optical, semiconducting, and electrical properties. These two nanomaterials can both help achieve high sensitivity and selectivity in biosensors and are also suitable for conjugation with biomolecules because of their excellent biocompatibility. Moreover, in recent years, studies have been conducted to create biosensors with graphene/MoS_2_ nanohybrids to achieve synergistic effects from each nanomaterial.

This review discusses recently reported biosensors based on graphene, MoS_2_, and graphene/MoS_2_ nanohybrids. The discussion was organized into four categories. First, the properties of graphene and MoS_2_, the synergistic effects derived from the conjugation of graphene and MoS_2_, and their advantages for developing the biosensors were provided. Next, graphene-based biosensors were discussed that employ an excellent electron transfer facilitation, quenching, and chemical enhancement effects for developing biosensors. Then, MoS_2_-based biosensors were described. These biosensors utilize the unique characteristics of the MoS_2_ in various structures, such as its luminescence and electrochemical properties. Finally, recent biosensors based on a graphene/MoS_2_ nanohybrid were discussed. These biosensors incorporated various structures of the graphene/MoS_2_ nanohybrid, which provide the advantages of each material and induce exceptional synergetic effects. Although various types of graphene/MoS_2_ nanohybrid have shown potential for biosensing application, there are still issues to be solved for practical or future applications. Above all else, a uniform mass production, the commercialization of various types of graphene/MoS_2_ nanohybrid, and reducing the production cost are essential for conducting efficient researches. Nevertheless, it is clear that a graphene/MoS_2_ nanohybrid is one of the most advanced nanomaterials for next-generation biosensors. In addition, through research to find more efficient types or structures of graphene/MoS_2_ nanohybrids, these can be used in wearable biosensors or applied to commercially available POCT. In conclusion, the recent research covered in this review, based on graphene/MoS_2_ nanohybrids, provides a new approach and interdisciplinary knowledge to advance the development of highly sensitive and selective biosensors.

## Figures and Tables

**Table 1:** Comparison and characteristics of graphene, MoS_2_, and the graphene/MoS_2_ nanohybrid utilized in highly sensitive biosensors.

Biosensors Based on the Graphene, MoS_2_ and Graphene/MoS_2_ Nanohybrid for Biosensors					
Type	Composition	Sensing Probe	Target	Utilized Technique	Reference
Graphene-based biosensors	Peptide/GO/AgNP	Peptide	PSA	Linear sweep voltammetry	[88]
	GO/AuNS	GO	Bilirubin	SERS	[96]
	BQD	BQD	Fe^3+^, Cyt C, Pi	Fluorescence	[104]
MoS_2_-based biosensors	Au/MoS_2_/Au multilayer/gp120 antibody	Gp120 antibody	gp120	Square wave voltammetry	[122]
	MoS_2_ nanosheet/ssDNA with FAM/DSN	ssDNA with FAM	let-7b miRNA	Fluorescence	[125]
	MoS_2_ QD/aptamer-modified AuNP	Aptamer	MC–LR	Fluorescence	[130]
Graphene/MoS_2_ nanohybrid-based biosensors	Mb/GO@MoS_2_	Mb	H_2_O_2_	Amperometry	[30]
	graphene QD/aptamer/MoS_2_ nanosheet	EpCAM aptamer	EpCAM	Fluorescence	[76]
	W-MoS_2_/ GMFs/3D nickel	Raman signal itself from each target molecule	rhodamine B, methylene blue and adenosine	SERS	[77]

## Data Availability

No new data were created or analyzed in this study.
